# Cardiovascular effects of *Adonis aestivalis *in anesthetized sheep

**Published:** 2014

**Authors:** Masoud Maham, Farshid Sarrafzadeh-Rezaei

**Affiliations:** *Department of Clinical Sciences, Faculty of Veterinary Medicine, Urmia University, Urmia, Iran*

**Keywords:** *Adonis aestivalis*, Cardiac arrhythmias, Electrocardiogram, LD_50 _

## Abstract

*Adonis aestivalis *(summer pheasant-eye) is an annual plant with a crimson flower, distributed in southern Europe and Asia. The plant has large buttercup-like blossoms and soft, fern-like leaves. It blooms in spring and is often found as a weed in cereal fields. Like other* Adonis *spp., the plant produces cardiac glycosides. It is used in remedies for mild weakness of the heart, especially when accompanied by nervous complaints. Cardiovascular and toxic effects of a hydroalcoholic extract from the aerial parts of *A. aestivalis* were investigated in sheep and mice. Six male sheep were anesthetized with sodium pentobarbital and arterial blood pressure was measured with a transducer connected to the left femoral artery. Heart rate and electrocardiogram (ECG) were registered from lead base-apex ECG derivatives connected to a Powerlab recorder. Three successive equal doses (75 mg kg^-1^) of the hydroalcoholic extract of *A. aestivalis* intravenously administered to anesthetized sheep. *Adonis aestivalis* extract induced a significant bradycardia and hypotension in sheep. Various ECG abnormalities in sheep included sinus arrhythmia, shortened and depressed S-T interval, and absence of P wave and flattened or inverted T wave. In addition, ventricular arrhythmias, bradyarrhythmias, atrioventricular block, ventricular premature beats, ventricular tachycardia and ventricular fibrillation have also been observed. The acute intraperitoneal toxicity (LD_50_) of the extract in mice was 2150 mg kg^-1^. In conclusion, bradycardia and ECG alterations induced by the extract could explain the justification of traditional use of the of *Adonis aestivalis* in treating cardiovascular insufficiency.

## Introduction

Since Sir William Withering suggested that digitalis may be beneficial in patients with heart complaints in 1785, they have now been used for medicinal purposes for more than 200 years. Today, digoxin and digitoxin are the only cardiac glycosides commercially available for prescription. Cardiac glycosides are, however, widely available in some botanic products and other naturally occurring substances.^1^

Cardiac glycosides have shown a large potential therapeutic effects, such as antiparasitic,^2^ antimicrobial,^3^ anti-HIV,^4^ and a significant anticancer activity.^5^ Cancer affects thousands of people and drug therapy is fundamental to increase survival or total cure of it. Recent reviews have highlighted the novel therapeutic applications of cardiac glycosides, in particular the accumulating *in vitro* and *in vivo* studies reporting antitumor activity. The first generation of glycoside-based anticancer drugs is in clinical trials.^6^

**Fig. 1:**
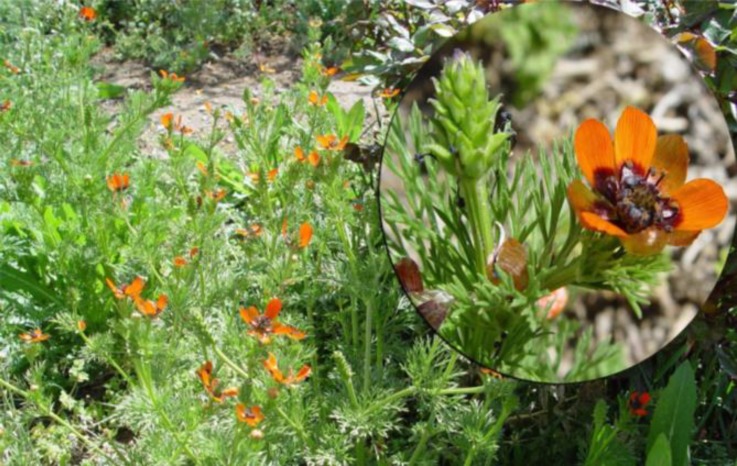
Leaves, flowers and fruits of *Adonis aestivalis*

*Adonis aestivalis* (summer pheasant's eye) is an annual plant with herbaceous growth, which grows about 40 cm tall and flowers from May to August with dark red blooms with dark centers and has linear threadlike leaflets (Fig. 1). Even though *A. aestivalis* has lower concentrations of cardiac glycosides than the false hellebore (*A. vernalis*), it remains a poisonous plant and should only be used medicinally under medical supervision. Infusions of *A. aestivalis* are used as diuretic, sleeping draught, and cough medicine. Species of *Adonis* are used to create medicines for stimulating heart function. The substance used is similar to those of *Digitalis* (foxglove) and are often prescribed in its place, to avoid the long-term effects of digitalis-derived drugs.^7^

According to the previous phytochemical study, the aerial parts of *A. aestivalis* contain four cardenolides.^8^ Seeds of *A. aestivalis* were reported to contain new cardenolide (3β, 5α, 14β, 17β-tetrahydroxycard-20, 22-enolide), two new glycosides, strophanthidin hexaglycoside and strophanthidin 3-O-β-D-glucopyranoside.^9^

Despite its use in traditional medicine, no data on the biological activity of the aerial parts of *A. aestivalis* are available. The claim of therapeutic success of the plant in the treatment of cardiovascular diseases has not been scientifically scrutinized. This prompted us to study the putative cardiovascular effects of a hydroalcoholic extract of this species in anesthetized sheep, as well as to evaluate its general toxic effects.

## Materials and Methods

The fresh and uncrushed aerial parts of *A. aestivalis* were collected in West Azerbaijan county (Urmia), Iran and were authenticated at Taxonomy Unit, Basic Science College of Tarbiat Modares University, Tehran, Iran. Whole plants were dried and ground in a hammer mill then were soaked in 70% ethanol and kept at room temperature for seven days. To complete extraction, it was mixed twice daily. This procedure was repeated four times and combined ethanolic extract was evaporated to dryness on a rotary evaporator maintaining the temperature between 45 and 50 ˚C, and a deep dark brown semi-solid residue was obtained. The pure *A. aestavalis* hydroalcoholic extract (AAHE) was kept protected from light in a refrigerator at 4 ˚C. We found out that the yield of extract was about 9.25%. The extract was dissolved in saline for evaluation of biological activities.

Animals. The experimental protocol was approved by the Experimentation Ethics Committee on Animal Use of the Faculty of Veterinary Medicine, Urmia University, Urmia, Iran. Healthy albino mice of either sex, weighing 18-33 g, were obtained from Pasture Institute, Tehran, Iran. The animals were housed three per cage, and the photoperiod (light on from 06:00 to 18:00 hr), air circulation and room temperature (24 ± 1 ˚C) were controlled. All animals had free access to tap water and standard rodent diet.

Six healthy, adult, mixed breed male sheep with a mean body weight of 33 kg (range: 30 to 37 kg) were used in this study. All sheep were judged to be healthy based on physical examination, complete blood count, biochemistry profile and electrocardiographic (ECG) examination. The sheep were fed hay concentrate twice daily, and had access to water *ad libitum*. Animals were conditioned to remain in lateral recumbency.

Acute toxicity and behavioral activity tests in mice. Pilot tests were conducted to determine the dose rang of the extract to be administered in mice. The maximum dose of AAHE, producing no death and the minimum dose that produced 100% death were achieved. From these, appropriate concentrations of extract dissolved in saline containing 0.1, 1.6, 2.9, 3.5 and 5.0 g kg^-1 ^of AAHE were given intraperitoneally (IP) to 6 groups of 5 mice each. The animals were observed for symptoms of toxicity and mortality within 24 to 72 hr. The LD_50_ was calculated based on Lorke’s method.^10^ Observation continued for 14 days to confirm that the number of animals per dose that remained alive did not alter. The behavioral and CNS profiles scored were: spontaneous rearing and grooming, evidence of calmness and sedation, loss of writhing reflex and duration of sleep.

Measurement of blood pressure (BP) and electrocardiography in anesthetized sheep. All sheep were anesthetized by first administering 30 mg kg^-1 ^of 5% solution of sodium pentobarbital (Sigma, St. Louis, MO, USA) intravenously, followed by small supplemental doses as needed. 

The left femoral artery was cannulated with poly-ethylene tubing PE-50 (Clay Adams, Parsippany, NJ, USA) filled with heparinized saline (60 IU mL^-1^), which was connected to a pressure transducer (Model MLT844; ADInstruments, Sydney, Australia) coupled to a bridge amplifier (Model ML224; ADInstruments, Sydney, Australia). The pressure transducers were calibrated with a medical manometer prior to each study, with the zero level set at the thoracic inlet of the laterally recumbent sheep. Mean arterial pressure (MAP) was calculated from the BP data sampled in an off-line analysis as:

*Blood pressure = diastolic + [(systolic - diastolic)/3]*

For continuous monitoring of the base-apex leads electrocardiogram, positive electrode of lead I (left arm) was attached to the skin of the left thorax at the fifth intercostal space immediately caudal to the olecranon, and the negative electrode (right arm) was placed on the jugular furrow in the caudal third of the left neck. The electrodes were placed using alligator clips and a gel contact, after clipping of the skin and cleaning with alcohol.^11^ The BP and ECG data were continuously displayed and recorded on-line on a personal computer by use of a data acquisition system (Model ML870; PowerLab, ADInstruments, Sydney, Australia) using LabChart (Version 6; ADInstruments, Sydney, Australia). Heart rate (HR) was calculated over the measurement period from a simultaneously recorded electrocardiogram. 

The left jugular vein was cannulated with similar tubing to facilitate intravenous injections of the drugs and plant material. The exposed surface for the cannulation was covered with cotton wool moistened in warm saline. 

After completion of surgical preparations, the sheep were allowed to stabilize for 20 min without further intervention. The baseline ECG and hemodynamic parameters were obtained 5 min before AAHE injection (control group). Repeated doses (3 doses, 75 mg kg^-1^, iv) of AAHE were injected into the jugular vein to assess their cardiovascular effects on anesthetized sheep. Arterial blood pressure was allowed to return to the resting level between injections. Changes in blood pressure were recorded as the difference between the steady state values before and the peak readings after injections.

For statistical analysis, significance differences between control and experimental groups were assessed by repeated measure ANOVA using SPSS (Version 17; SPSS Inc., Chicago, USA). Results were considered significant when *p* < 0.05. Data are expressed as mean ± standard error of mean (SEM).

## Results

**Acute toxicity in mice.** The mortality rate of the intraperitoneally administered AAHE increased progressively with the increasing dose (data not shown): the mortality rate of 0% at and up to a dose of 1500 mg kg^-1^ gradually rose to 100% at 5000 mg kg^-1^, the highest dose studied. The no-observed-adverse-effect level (NOAEL) for the intraperitoneal dose was 1000 mg kg^-1^, while the lowest-observed-adverse-effect level (LOAEL) was 1600 mg kg^-1^. The severity of clinical signs was similar appreciably between individuals. Some adverse effects, such as salivation, hypo-activity, ataxia, posterior paralysis and recumbency were seen immediately after the intraperitoneal injection, while others (decreased appetite and weight loss) were observed soon after, and were more pronounced at the higher doses. Interestingly limb paralysis that leads to recumbency was resolved spontaneously later. The acute intraperitoneal toxicity (LD_50_) of AAHE in mice was 2150 mg kg^-1^.

**Fig. 2:**
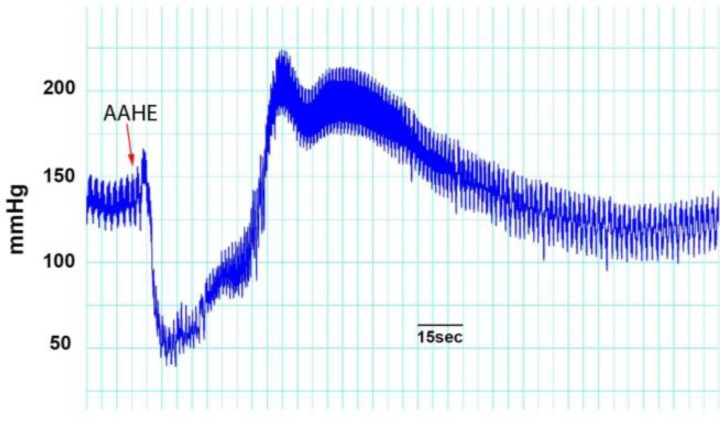
Effect of AAHE on blood pressure in anesthetized sheep

**Fig. 3:**
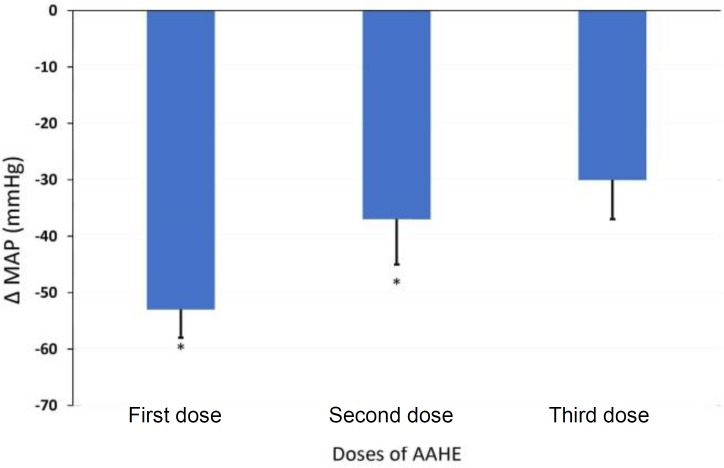
Effect of 3 doses of hydroalcoholic extract of *Adonis aestivalis* (AAHE; 75 mg kg^-1^, intravenous) on mean arterial pressure of anesthetized sheep. Data represent the mean ± SEM of six animals. * indicates significant different compared to control group at *p* < 0.05.

**Fig. 4:**
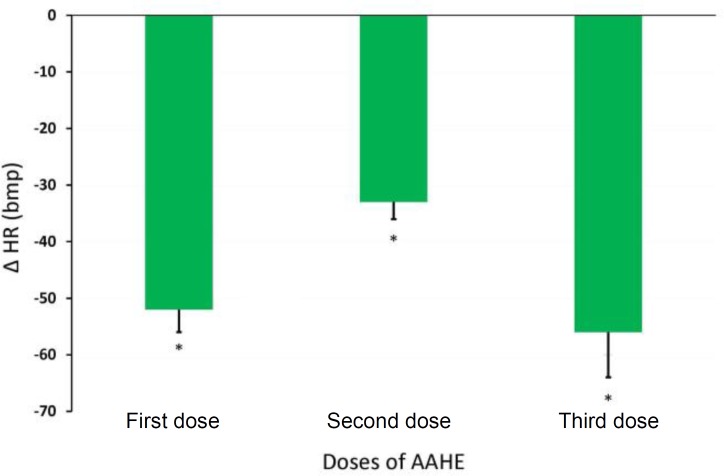
Effect of 3 doses of hydroalcoholic extract of *Adonis aestivalis* (AAHE; 75 mg kg^-1^, intravenous) on heart rate of anesthetized sheep. Data represent the mean ± SEM of six animals. * indicates significant different compared to control group at *p* < 0.05

**Effects of AAHE on MAP and HR.** The intravenous injections of AAHE (75 mg kg^-1^) produced a reproducible reduction in MAP and HR. Acute administration of AAHE caused a rapid-onset decrease in MAP (Fig. 2) and HR, while the hypotensive and bradycardic effects were rather short-lived and returned toward the baseline levels within 2 to 5 min after first dose administration. A more pronounced and long-lasting response was observed at the injection of second dose and both MAP (Fig. 3) and HR remained depressed even at 5 min after AAHE administration (Fig. 4). In all of the experiments, third dose caused a rapid increase in mean blood pressure for few minutes; followed by a progressive and irreversible fall in MAP.

**Fig. 5:**
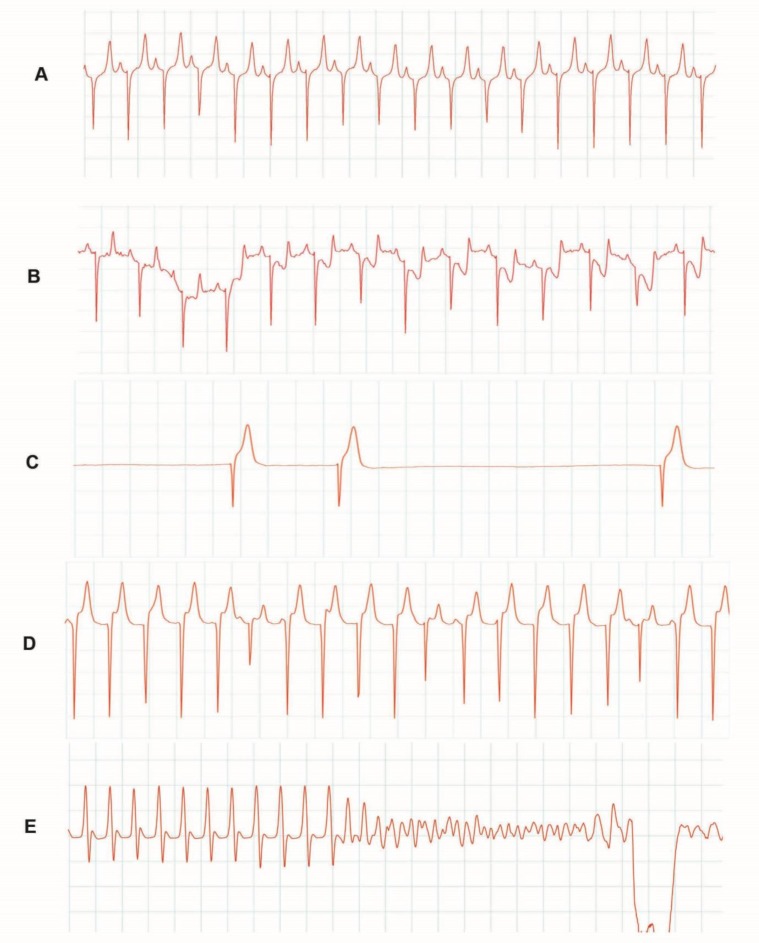
Typical ECG changes indicating arrhythmia indexes in anesthetized sheep. **A.** ECG before the application of AAHE. The changes observed after the application of extract include: **B.** ST depression; **C.** Escape beats; **D.** Idioventricular rhythm and **E.** Ventricular fibrillation

**Effects of AAHE on electrocardiogram.** Heart rate was reduced 2 to 4 min after first AAHE administration significantly (*p *< 0.05). There were no significant alterations in duration and amplitude of P wave, QRS complex and T wave. P-R interval, Q-T interval and S-T segment did not show important changes during this period (*p* < 0.05). However, in all animals second dose produced a significant change in P-R and Q-T intervals. Bradycardia was replaced with pauses and tachyarrhythmias in third AAHE injection. Various ECG abnormalities observed in sheep included sinus arrhythmia, shortened and depressed S-T interval, absence of P wave and flattened or inverted T wave. Additionally, ventricular arrhythmias, bradyarrhythmias, atrioventricular (AV) block, ventricular premature beats (VPB), ventricular tachycardia (VT) and ventricular fibrillation (VF) have also been observed (Fig. 5). 

## Discussion

Hypoactivity, ataxia, posterior paralysis and recumbency observed in mice in this study as poisoning symptoms are strong indications of other sites of activity of the extract of *A. aestivalis* besides the heart and could be manifestations of damage done to the heart and/or the central nervous system. In reference survey, we did not find any experimental study related to this subject, however, Zia *et al*. reported that two purified fractions (B1 and B3) obtained from the methanol extract of fresh oleander leaves (another plant containing cardenolides) possess a CNS depressant activity, i.e. produced reduction in locomotor activity, rotarod performance and potentiate hexobarbital-induced sleeping time.^12^

Our study shows for the first time that AAHE has marked pharmacological effects on the cardiovascular system, inducing bradycardia, various dysrhythmias (particularly AVB, VT and VF) and producing a transient fall of blood pressure in anesthetized sheep. 

The most active components of AAHE are cardiac glycosides (cardenolides) similar in structure to digoxin.^7^^-^^9^ The present data showed that intravenous administration of AAHE induced significant negative chronotropic effect (bradycardia) in anesthetized sheep. The reported negative chronotropic effect of AAHE is similar to that of digitalis cardiac glycosides. It is well known that digitalis cardiac glycosides exert a number of effects on the neural tissue that indirectly influences the mechanical and electrical activities of the heart. Digitalis increases the para-sympathetic activity of cardiovascular system and inhibits its sympathetic terminals leading to bradycardia.^13^

Several cases of cardiac disorders such as sinus arrhythmia, junctional ectopic beats, negative QRS deflection, ventricular tachycardia, and heart block were recorded (Fig. 3). Sinus arrhythmia may occur because the sinus node does not discharge with absolute regularity due to variations in vagal tone related to the respiration.^14^ Moreover, the results of this investigation indicated that AAHE affected greatly the site of origin of cardiac impulses, and there was alteration in the direction of the excitation waves from the normal pacemaker to another focus in the conductive system. This is manifested by several cases of junctional ectopic beats. It is believed that escape rhythms are not primary disorders, but are protective mechanisms against the failure of SA node depolarization or the conduction block.^15^

The arrhythmogenic effects of the cardiac glycosides are due to a combination of direct effects on the myocardium and conducting system of the heart, and neurally mediated increases in autonomic activity.^16^ Cardiac glycosides bind to cardiac muscle plasma membrane Na^+^-K^+^-ATPases, leading to a decrease in the net cellular uptake of K^+^ and a rise in the intracellular Na^+ ^concentration. The rise in intracellular Na^+^ concentrations causes intracellular Ca^2+^ overload because of the reduction of Ca^2+^ efflux via the Na^+^/Ca^2+^ exchange system. This Ca^2+^ overload in turn induces oscillatory Ca^2+^ release from the sarcoplasmic reticulum and oscillatory fluctuation in resting membrane potential. An ionic current associated with this Ca^2+^ oscillation is known as the transient inward current. It has been reported that about 75.00% of the transient inward current is caused by an ionic current generated by Na^+^/Ca^2+^ exchange while the remaining current is mediated through nonspecific cation channels.^17^

A prominent hypothesis to explain the cardiotoxicity of AAHE is the accumulation of large amounts of calcium in myocardial cells which may alter the integrity and function of several membrane systems and affect mitochondrial energy production. Oxidative degeneration of membrane lipids may result in increased sarcolemmal calcium permeability and alter calcium regulatory mechanisms.^18^

Our results also indicated that a decrease of blood pressure occurred simultaneously with the beginning of the AAHE-induced bradycardic effect.

However, blood pressure rapidly returned to normal, despite the persistence of bradycardia. This transient effect of AAHE on blood pressure might have been due to a vaso-constrictor effect of AAHE. There exist some studies that show the vasoconstrictor activity of cardiac glycosides.^19^

To the best of our knowledge, this is the first study investigating the direct effects of AAHE on the cardiovascular system through *in vivo* experiments. Natural toxicoses of *A. aestivalis* is documented in horses. Gastrointestinal stasis and myocardial necrosis were reported in horses after consuming hay contaminated with *A. aestivalis*.^20^ In a previous study, sheep did not develop clinical signs when dosed with 1% body weight ground *Adonis* (estimated content 11 to 17 ppm strophanthidin) via rumen cannulas, or when dosed daily for two weeks with 0.2% bodyweight *Adonis* per day. Extensive cardiac examinations demonstrated transient cardiac functional effects. No gross or microscopic lesions were evident in the heart or any other tissues examined. The authors concluded that sheep are resistant to intoxication after ingestion of reasonable quantities of *A. aestivalis*, identifying sheep as potential livestock species to which contaminated hay could be fed safely.^21^ Their results were different to those of our study that can be explained in relation with the route of administration and gastrointestinal degradation of cardiac glycosides. The effects of several poisons may vary to a considerable extent in polygastric versus monogastric species. First of all, the considerable volume of ruminal fluid (100 to 225 L in cattle and 10 to 25 L in sheep and goats) leads to a dilution of toxic materials decreasing their absorption rates. Second, and most importantly, ruminal microorganisms carry out a number of biotransformation reactions, mainly hydrolysis and reduction responsible for the detoxification of poisonous substances.^22^

On the other hand, these dissimilar results among the same species are probably related to variations between different amounts of *Adonis* cardenolides resulting from various geographical areas and soil type. Furthermore, the climate and stage of plant growth may also cause variation in the toxicity.^23^

The noticed electrophysiological characteristics of AAHE either its negative chronotropic or ECG effects could have a promising and potential medicinal importance (like digoxin) for managing some heart diseases, especially in treatment of congestive heart failure which represented as impaired myocardial relaxation (diastolic dysfunction) or impaired myocardial contractility (systolic dysfunction). Further studies are required to clarify the ion selectivity and ion channels which are modulated by AAHE. 
